# High mutation burden in the checkpoint and micro-RNA processing genes in myelodysplastic syndrome

**DOI:** 10.1371/journal.pone.0248430

**Published:** 2021-03-17

**Authors:** Ivan Sergeevich Moiseev, Nikolay Yurevich Tcvetkov, Ildar Munerovich Barkhatov, Maria Vladimirovna Barabanshikova, Dmitrii Sergeevich Bug, Natalya Vitalievna Petuhova, Artem Valerievich Tishkov, Evgenyi Alexandrovich Bakin, Ekaterina Andreevna Izmailova, Alena Igorevna Shakirova, Alexandr Dmitrievich Kulagin, Elena Vladislavovna Morozova

**Affiliations:** 1 RM Gorbacheva Research Institute, Pavlov University, Saint-Petersburg, Russian Federation; 2 Bioinformatics Department, Pavlov University, Saint-Petersburg, Russian Federation; Beth Israel Deaconess Medical Center-Harvard Medical School, UNITED STATES

## Abstract

A number of sequencing studies identified the prognostic impact of somatic mutations in myelodysplastic syndrome (MDS). However the majority of them focused on methylation regulation, apoptosis and proliferation genes. Despite the number of experimental studies published on the role of micro-RNA processing and checkpoint genes in the development of MDS, the clinical data about mutational landscape in these genes is limited. We performed a pilot study which evaluated mutational burden in these genes and their association with common MDS mutations. High prevalence of mutations was observed in the genes studied: 54% had mutations in DICER1, 46% had mutations in LAG3, 20% in CTLA4, 23% in B7-H3, 17% in DROSHA, 14% in PD-1 and 3% in PD-1L. Cluster analysis that included these mutations along with mutations in ASXL1, DNMT3A, EZH2, IDH1, RUNX1, SF3B1, SRSF2, TET2 and TP53 effectively predicted overall survival in the study group (HR 4.2, 95%CI 1.3–13.6, p = 0.016). The study results create the rational for incorporating micro-RNA processing and checkpoint genes in the sequencing panels for MDS and evaluate their role in the multicenter studies.

## Introduction

Myelodysplastic syndrome (MDS) is a heterogenic group of diseases characterized by accumulation of somatic mutations [1–3], alterations in the bone marrow niches [4], various pathological events in the immune system, including pyroptosis and autoimmune bone marrow damage [5, 6], tumor escape at later stages [7] and ineffective hematopoiesis as a result of aforementioned events. Genome instability and high incidence of secondary cancerogenic genetic events determines frequent transformation of MDS to acute myeloid leukemia (AML) [8]. The current standard of care in high-risk MDS are hypomethylating agents [9, 10], which significantly improve time to progression and survival, but only in the minority of patients they induce complete remission (CR). The only curative option is an allogeneic stem cell transplantation (SCT), but even in candidates in the modern era of advanced supportive care the results are generally worse than in CR of acute leukemia with only 30–40% of overall survival in 5 years [11, 12].

The relatively unfavorable outcomes after existing therapies drive the search for a novel therapeutic targets in high-risk MDS. One of the breakthroughs in modern oncology is the introduction of the checkpoint inhibitors into clinical practice [13]. The analysis of the checkpoint proteins expression in the bone marrow of MDS and AML patients demonstrated that myeloid cells express different checkpoint ligands and receptors, including CD80, CD86 and PD-1L [14–16]. However, the best response observed in the clinical studies of checkpoint inhibitors was “stable disease”, despite the fact that some patients had stabilization for a long period of time, indicating the potential efficacy of these agents in MDS [17]. The other checkpoint inhibitors, like anti-TIM3 and anti-CD47, have a more promising response rate, but longer follow up is required to determine whether this response translates into long-term remission [18, 19].

Another aspect of MDS pathogenesis is the changes in a bone marrow niche [20, 21]. The experimental studies indicate that knock out of the genes involved in micro-RNA processing and extracellular signaling, like DICER1, DROSHA and SBDS may lead to MDS-like phenotype [22]. Expression profile of these genes is also altered in MDS [23]. Despite current studies with next-generation sequencing (NGS) that include thousands of patients, these studies focus on 36–55 genes related to methylation, proliferation and apoptosis, while full exome sequencing is generally used to validate the results of panel sequencing in the subgroup of patients [24]. Despite some studies do focus on micro-RNA processing [25, 26] and checkpoint genes [27] in solid tumors, data in MDS regarding these additional potential mechanisms of MDS progression and resistance is limited. Thus we performed a pilot study evaluating interaction of mutations in the most commonly mutated genes, checkpoint and micro-RNA-associated genes.

## Methods

### Patients

The study included 35 patients with high-risk MDS consulted at the hematopoietic stem cell transplantation (HSCT) center at the time of diagnosis during 2008–2018. All patients provided informed consent for the use of their biological material in the research purposes. A total of 48 samples from the 35 enrolled patients were analyzed. The study was approved by the Ethical committee of the First Pavlov Medical University and performed in accordance with the ethical standards laid down in the 1964 Declaration of Helsinki and its later amendments. All patients included in the study signed written informed consent for the use of their biological materials and medical records for research purposes before their inclusion in the study. The DNA samples were anonymized before sequencing. All samples, except longitudinal samples in six patients, were taken at diagnosis. The sequencing was performed in Apr 2020. The median age was 49 years (range 18–80). Eighty two percent had high or very high risk according to IPSS-R. Twenty five patients undergone HSCT the others received therapy with hypomethylating agents (Table 1).

**Table 1:** Characteristics of patients.

Parameter	N, (%)
Age, years, median (range)	49 (18–80)
Gender	
males	21 (60%)
females	14 (40%)
IPSS-R score	
Low	1 (3%)
Intermediate	5 (14%)
High	18 (51%)
Very high	11 (31%)
Blasts in bone marrow	
0–4.9%	8 (23%)
5–10%	16 (46%)
10.1–20%	11 (31%)
Karyotype	
Normal	11 (31%)
Monosomal	6 (17%)
Complex	5 (14%)
Other	13 (37%)
Neutrophils, median (range), 10^9^/L	1.0 (0–9.0)
Platelets, median (range), 10^9^/L	90 (10–518)
Transfusion dependence	9 (26%)
SCT performed	19 (54%)

### Targeted sequencing

Genomic DNA was extracted from the fresh bone marrow aspirates using TriZ reagent extraction Kit (Inogene, Russian Federation) and stored at -80°C until the day of the assay. Whole bone marrow was used to potentially capture the mutations in the bone marrow niche cells. Separation of subpopulations of cells was not performed before sequencing. The quality of the samples before the assay was analyzed using Qubit 4.0 (Thermo Fisher, CA, USA).

The libraries for target sequencing of genes were prepared using shotgun liquid hybridization method with DNA probes. KAPA HyperPlus Kit (Roche, Switzerland) according to the manufacturer instructions were used for library preparation. The enrichment of targeted genome sequences was performed using SeqCap EZ Target Enrichment System (Roche, Switzerland) according to the manufacturer instructions. The enrichment of the coding sequences was performed for the following genes: ASXL1, CD274 (Programmed cell death 1 ligand 1), CD276 (В7-Н3 ligand to CTLA4 and CD28 receptors), DICER1, DNMT3A, DROSHA, EZH2, IDH1, IDH2, LAG3, MFSD11 (Major facilitator superfamily domain containing 11), PDCD1 (Programmed cell death 1 receptor), PIKFYVE (Phosphoinositide kinase, FYVE-type zinc finger containing), RUNX1, SF3B1, SRSF2, TET2 and TP53. The coverage of the target genes was at least x1000. Sequencing was performed with MiSeq using MiSeq Reagent Kits v2 (Illumina, USA) with 2х250 n.p. complementary read regimen. The primary reads are available at BioProject, accession number PRJNA631513, https://www.ncbi.nlm.nih.gov/bioproject/631513.

### Bioinformatics

Quality of reads was assessed using FastQC [28], they were aligned to the GRCh38 reference genome with BWA [29], next GATK 4.1.5.0 [30] duplicate marking, sorting, and base quality score recalibration tools were applied. SNP and INDEL calling was performed using Mutect2 algorithm [31] according to GATK Best Practices recommendations [32]. Effects of discovered variants was determined by Ensembl Variant Effect Predictor [33] and ANNOVAR [34], using RefSeq annotation [35], population and variant interpretation databases (COSMIC [36], GnomAD [37], ClinVar [38]), and prediction tools (PolyPhen-2 [39], SIFT [40], MutationTaster2 [41], MutationAssessor [42], PROVEAN [43], and CADD [44]). Variants with allele frequencies more than 1% according to GnomAD data were filtered.

### Statistical analysis

The set of single nucleotide polymorphisms (SNPs) obtained with GATK was filtered according to the functionality and loci (synonymous, intronic and intergenic items were removed). The variant allele frequency (VAF) threshold of 5% was chosen to describe the frequency of common MDS-related mutations as the most frequently used presentation of the data. However the general analysis of mutation frequency in the microRNA processing genes and checkpoint genes was carried out with the 1% threshold, because these genes were described to have significant impact on microenvironment cells and tumor-infiltrating macrophages that comprise usually minor populations. The 1% threshold was supposed to capture these minor subpopulations. On the other hand, it ensured at least 10x reads per each mutation detected to avoid false positive results. Since the clinical relevance of the mutations in the microRNA processing and checkpoint genes is not determined, all mutations, exon and UTR, were included in the analysis. For clustering analysis those SNPs that were detected in only one patient, were excluded. In clustering analysis an every remained SNP we obtained a typical AF in the sample: a median AF among all the patients was calculated and rounded to the closest value from the set {0%, 50%, 100%}. This value was subtracted from every particular patient AF, thus providing an individual frequency shift bounded between -100% and +100%. A matrix of frequency shifts was composed, in which rows represented SNPs, and columns represented patients. A tree clustering was performed for the matrix columns and rows (Euclidian distance was used as a measure of items similarity). Top two patients clusters were taken for the downstream analysis: a set of clinical parameters were compared between clusters as well as survival characteristics. Quantitative parameters were compared with a Mann-Whitney test, survival analysis was performed with Kaplan-Meyer method. Heatmap was processed and visualized with *pheatmap* [45]. Circos plot was implemented with *circlize* package [46]. TCGAbiolinksGUI was used to visualize association of mutations [47].

## Results

### Identified mutations

**Fig 1:**
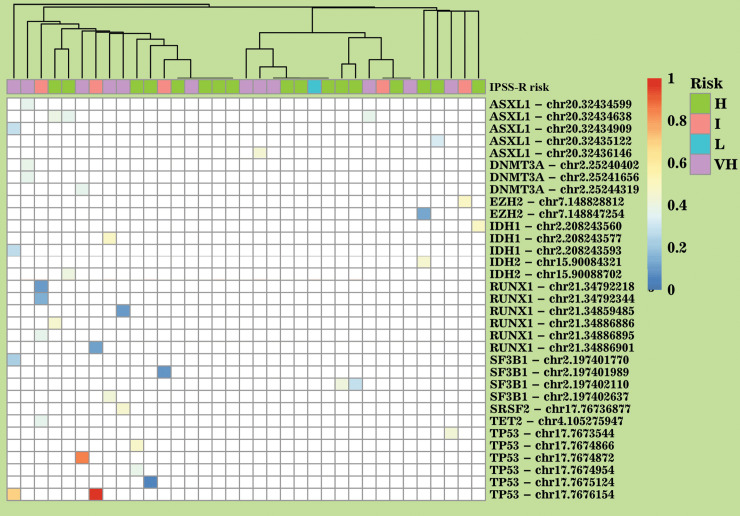
Prevalence of common exonic pathogenic mutations in the study group. White color in the heatmap represents absence of mutations. Blue colors represent mutations with VAF<50%, orange and red with VAF>50%. The risk line is the IPSS-R score presented by groups: low (L), intermediate (I), high (H), very high (VH). Transplantation line indicates whether the patient was allografted. (*) indicates mutations which are present in the COSMIC database. (**) indicates mutations associated with oncohematological diseases in the COSMIC database.

The pattern of common MDS mutations with ≥5% VAF was similar to the previous studies. Twenty percent of patients at diagnosis had mutations in the ASXL1, 17% in TP53, 14% in DNMT3A, 14% in SF3B1, 11% in RUNX1, 9% in IDH1 and 6% in IDH2 and EZH1 each. Single instances of TET2 and SRFS2 were indentified. No common mutations were found in 14% of patients (Fig 1, S1 and S2 Figs).

Since there is no data about the pathogenic impact of mutations in the micro-RNA processing genes and checkpoint genes as well as the size of clinically relevant populations of cells with mutations the analysis selected all mutations that affected either protein sequence or modified gene expression. Polymorphisms effecting more than 50% of patients were not accounted, but displayed in the graphical form. In the studied set of genes 140 unique mutations were indentified that fit the selection criteria (S1 Table). A significant number of mutations was observed in the checkpoint genes: 46% of patients had mutations in LAG3, 20% in CTLA4, 23% in B7-H3, 14% in PD-1 and 3% in PD-1L. Also a significant number of patients with PD-1L polymorphisms previously described in relation to adverse cancer outcomes were determined: rs4742098 in 54%, rs2297136 in 63%, rs4143815 in 54%. No previously described polymorphisms in other genes were identified.

Furthermore the prevalence of mutations in the micro-RNA processing genes was also relatively high: 17% in DROSHA and 54% in DICER1. A number of polymorphisms in these genes were also high but their significance is still undetermined. The cumulative incidence of SNPs in the studied set of genes regardless of their established pathogenic impact was highest in ASXL1, TET2, DICER1 and RUNX1 (S3 Fig).

The majority of observed SNPs are not reported to be pathogenic, so the following steps were performed to evaluate the influence of mutations on the clinical course of the disease: 1) deviations in VAF from the median in the group were calculated. This step allowed to separate both patients with abnormal polymorphisms and clonal changes; 2) tree clustering analysis was performed based on these deviations from median VAF in the group; 3) two clusters were identified (S4 Fig). Since the multiple comparison correction did not allow to achieve statistically significant results in the frequency of certain mutations, they were ordered by descending significance of differences between clusters. The first 15 most significant mutations demonstrated that in cluster 1 there was a higher prevalence of SF3B1, less DICER1 mutations with high VAF and less B7-H3 (CD276) mutations. Cluster 2 harbored more ASXL1 mutations, RUNX1 mutations, more PD-1L (CD274) mutations.

**Fig 2:**
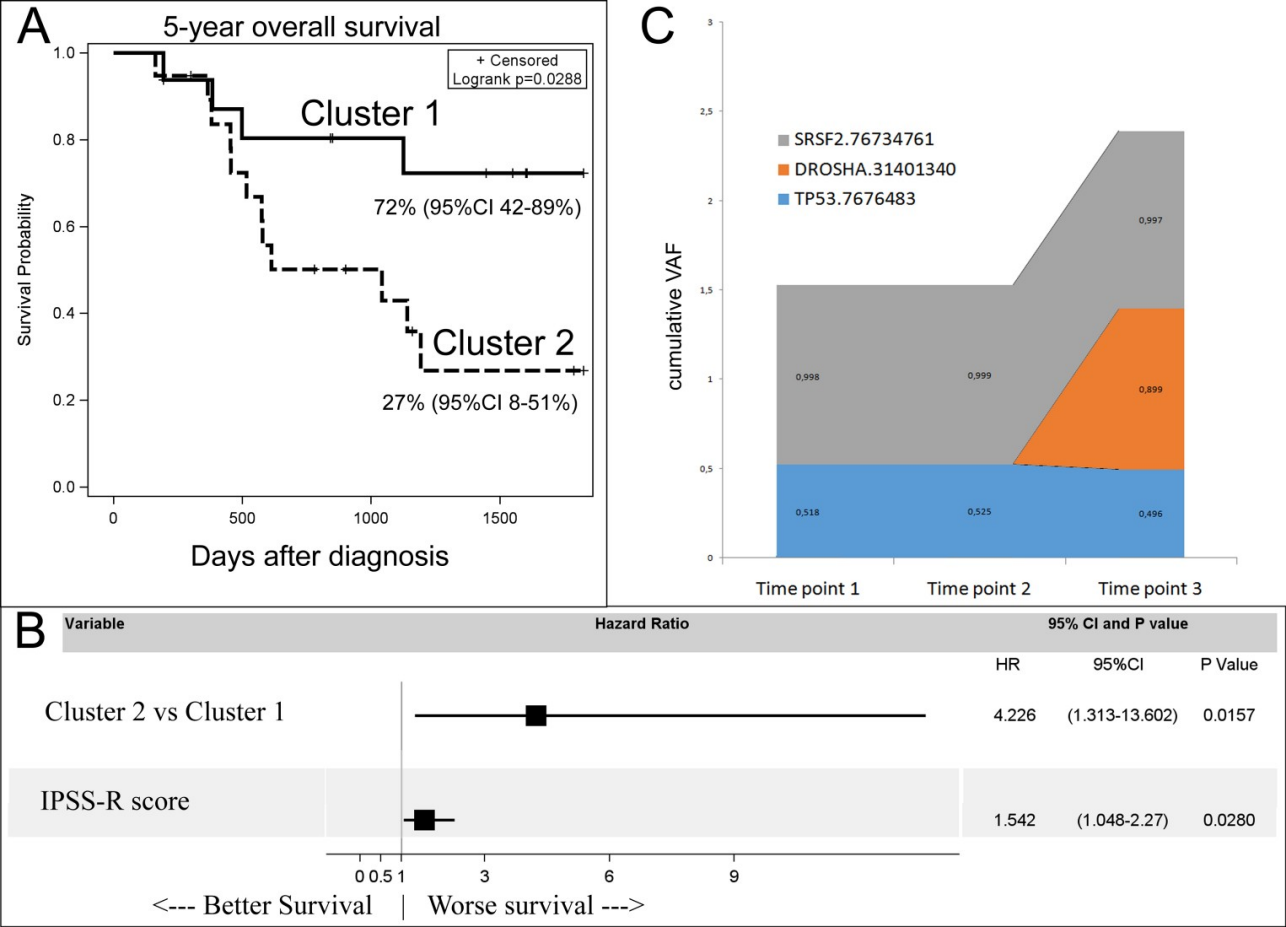
A. 5-year overall survival of patients in the identified clusters. B. Forrest plot with multivariate analysis of genetic clusters and IPSS-R. C. Example of clonal evolution with microRNA processing gene.

There was no difference between the identified clusters in the rate of SCT performed (50% vs 56%, p = 0.75). There was also no association of identified mutation clusters with IPSS-R score (p = 0.58), WPSS score (p = 0.34), Armand et al score (p = 0.21), age of the patients (p = 0.43), percentage of blasts in the bone marrow at diagnosis (p = 0.2), hemoglobin level at diagnosis (p = 0.84) and platelet level at diagnosis (p = 0.085). Also the distribution of patients who received HSCT was not different between two clusters (69% vs 73%, p = 0.75). Thus, the fact of HSCT did not interfered in the results of the analysis. There was a week association with neutrophil levels at diagnosis. Patients in the cluster 1 had lower levels (median 670 vs 990 x10^9/L, p = 0.013) (S5 Fig). Nonetheless there was a significant difference in overall survival. The 5-year overall survival estimate was higher in cluster 1 patients: 72% (95%CI 42–89%) vs 27% (95%CI 8–51%), p = 0.029 (Fig 2A). In the multivariate analysis with correction for IPSS-R score (HR 1.5, 95%CI 1.0–2.3, р = 0.28), the clusterization remained a significant predictor of all-cause mortality (HR 4.2, 95%CI 1.3–13.6, p = 0.016, Fig 2B).

**Fig 3:**
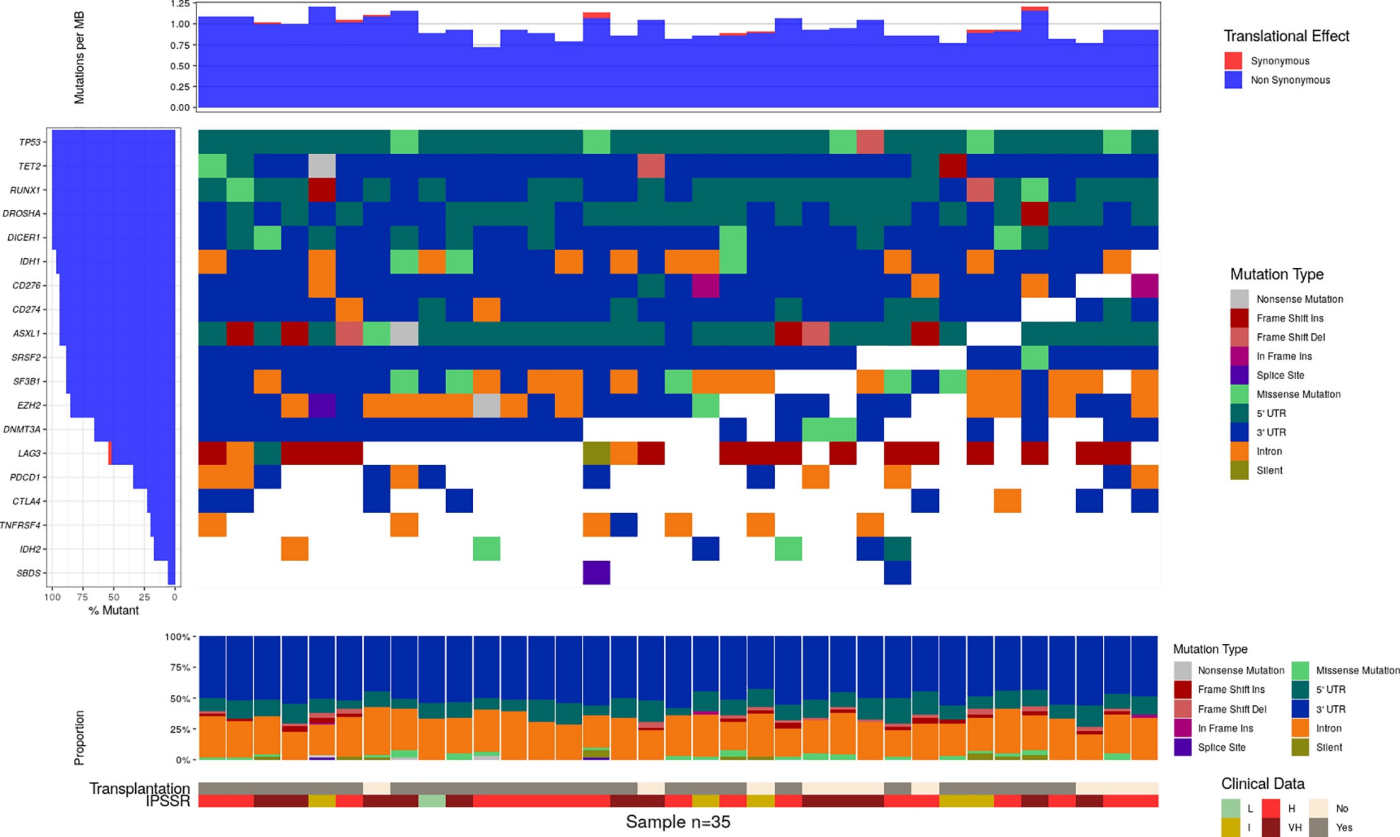
Associations between genetic abnormalities in the individual patients. The left vertical bar indicate the percentage of patients with abnormalities in the gene. Red color indicate synonymous substitution. Upper horizontal bar characterize mutation burden in the individual patients per megabase pair. Middle boxplot indicates that type of mutations in the genes in individual patients. Mutations are ordered and presented based on their deleterious effect in the order as shown in the legend. Additional less deleterious mutations in the gene are not shown. Horizontal lower bar plot indicate the type of genetic alterations present in individual patient across all genes tested. The risk line is the IPSS-R score presented by groups: low (L), intermediate (I), high (H), very high (VH). Transplantation line indicates whether the patient was allografted.

The analysis of mutation associations demonstrated uniform occurrence of mutations in the known MDS-associated genes and DICER1, DROSHA, and checkpoint genes. Except LAG3 with significant prevalence of missense mutations, SNPs in DICER1, DROSHA, CD274 and CD276 were predominantly documented in 5-UTR and 3-UTR regions (Fig 3, S6 Fig). The uniform distribution indicates that there is no pathogenic link between these mutations and they accumulate sporadically.

In seven patients who did have longitudinal samples of the bone marrow aspirate the clonal evolution was traced with the same set of genes. The mutations appearing during the disease coarse affected tp53, SRSF2, DROSHA and DICER1 with high AF in the studied patients. The other mutations in the checkpoint genes were observed in the minor clones with predominant involvement of LAG3 (S7 Fig).

## Discussion

MDS is the disease with one of the highest number of sequencing studies [48], however this is one of the first in MDS that confirmed the presence of mutations in checkpoint and micro-RNA processing genes and more than half of high-risk patients had these aberrations. This profile of mutations explains several observations from the experimental studies.

Both DROSHA and DICER1 are RNase III enzymes involved in the processing micro-RNA in the nucleus. It was demonstrated that alterations of micro-RNA signaling is due to abnormal functioning of these two enzymes [49]. In MDS multiple miRNAs were reported to be abnormally expressed, including pro-apoptotic miR-34a, anti-apoptotic miR-378 and miR-144, antioxidant miR-451 [50, 51], anti-DNMT1 miR-126 [52]. In mouse studies it was demonstrated that knock out of DICER1 in the mesenchymal cells in the bone marrow leads to abnormal expression of more than 10 miRNAs and MDS-like phenotype [53, 54]. Several miRNAs also regulate NLRP3 inflammasome which facilitates pyroptosis and hematopoiesis aging in MDS [5, 53, 54]. The observed VAFs and prevalence in the study group of DICER and DROSHA SNPs indicated that there were both polymorphisms and minor clones with somatic mutations, probably associated with the bone marrow niche cells. Further studies with selected subpopulations of cells are required to confirm the exact role of each genetic aberration in these genes.

Another aspect revealed in this study is the high frequency of mutations in the checkpoint genes. It was demonstrated that a number of checkpoint ligands, like PD-1L, PD-2L, B7, CD80 are overexpressed in MDS and in certain instances they are induced via inflammasome activation [55–57]. The accumulation of mutations in receptor genes leads to infective interaction with ligands and thus might represent the evolutionary protective changes against tumor progression in the setting of unstable genome and clonal hematopoiesis. The same finding may explain the moderate response to checkpoint blockade in MDS. The monoclonal antibodies may either not bind to the receptor with abnormal conformation or this receptor may not be expressed at all due to missense and frame shift mutations [57, 58].

The study does have several limitations, primary due to small number of patients. Particularly cautious should be the interpretation of clinical results. The difference in survival presented in the article was not to suggest the clinical predictive algorithm, but rather to demonstrate that when the results of the studied gene panel were analyzed mathematically there was some predictive power for the clinical outcomes. Also germline cells were not analyzed in parallel, which forced us to implement an advanced statistical methodology that facilitated interpretation of this data. However the major point of the study was not to identify and validate the significance of certain mutations, but rather highlight the importance of mutations in miRNA processing and checkpoint genes that should be included in the common MDS sequencing panels and evaluated in the large muticenter studies for their potential prognostic value and role in the pathogenesis.

## Supporting information

S1 Fig The heatmap of single nucleotide polymorphisms in the studied genes. The brighter colors indicate higher number of mutations in the patients’ genes. The risk line is the IPSS-R score presented by groups: low (L), intermediate (I), high (H), very high (VH).
(PDF)

S2 Fig Prevalence of common exonic pathogenic mutations leading to transcription failure. The left vertical bar indicate the percentage of patients with abnormalities in the gene. Upper horizontal bar characterize mutation burden in the individual patients per megabase pair. Middle boxplot indicates that type of mutations in the genes in individual patients. Mutations are ordered and presented based on their deleterious effect in the order as shown in the legend. Additional less deleterious mutations in the gene are not shown. Horizontal lower bar plot indicate the type of genetic alterations present in individual patient across all genes tested. The risk line is the IPSS-R score presented by groups: low (L), intermediate (I), high (H), very high (VH). Transplantation line indicates whether the patient was allografted.
(PDF)

S3 Fig Cluster analyses and heatmap of mutations in the study group. The heat map includes both pathogeneic mutations and SNPs with undetermined significance. The risk line is the IPSS-R score presented by groups: low (L), intermediate (I), high (H), very high (VH). Dark blue color represents high allele frequency shift downwards (up to -100%) from common allele frequency in the sample (the most part of the sample is homozygous with presence of this SNP, while the particular patient is homozygous with absence of this SNP). Similarly, red colors represent high allele frequency allele frequency shift upwards (the most part of the sample is homozygous with absence of this SNP, while the particular patient is homozygous with presence of this SNP). Yellow colors represent allele frequencies close to the median allele frequency for this gene in a patient. The patients are clustered according to their mutation patterns.
(PDF)

S4 Fig Prevalence of exonic and UTR-3 SNPs in the studied gene panel. White color in the heatmap represents absence of mutations. Blue colors represent mutations with VAF<50%, orange and red with VAF>50%. The risk line is the IPSS-R score presented by groups: low (L), intermediate (I), high (H), very high (VH). Transplantation line indicates whether the patient was allografted. (*) indicates mutations which are present in the COSMIC database. (**) indicates mutations associated with oncohematological diseases in the COSMIC database.
(PDF)

S5 Fig Non-parametric analysis of clinical characteristics at diagnosis in clusters 1 and 2. (PDF)

S6 Fig Associations between genetic abnormalities in the proportion of the study cohort with several mutated genes. Patients with only one mutated gene as well as genes with only one patient with mutation were excluded from analysis. The width of circus fragment represents the incidence of mutations in the specific genes. The width of the ribbon between the genes represents the rate of association.
(PDF)

S7 Fig Clonal evolution in the patients with longitudinal samples of bone marrow. (PDF)

S1 Table Description of identified genetic aberrations. (XLSX)
