# Supplementary material for: High mutation burden in the checkpoint and micro-RNA processing genes in myelodysplastic syndrome
Source: PLoS One. 2021 Mar 17;16(3):e0248430. doi: 10.1371/journal.pone.0248430 (PMC7968630; doi:10.1371/journal.pone.0248430)

S1 Fig. The heatmap of single nucleotide polymorphisms in the studied genes. The brighter colors indicate higher number of mutations in the patients' genes. The risk line is the IPSS-R score presented by groups: low (L), intermediate (I), high (H), very high (VH).

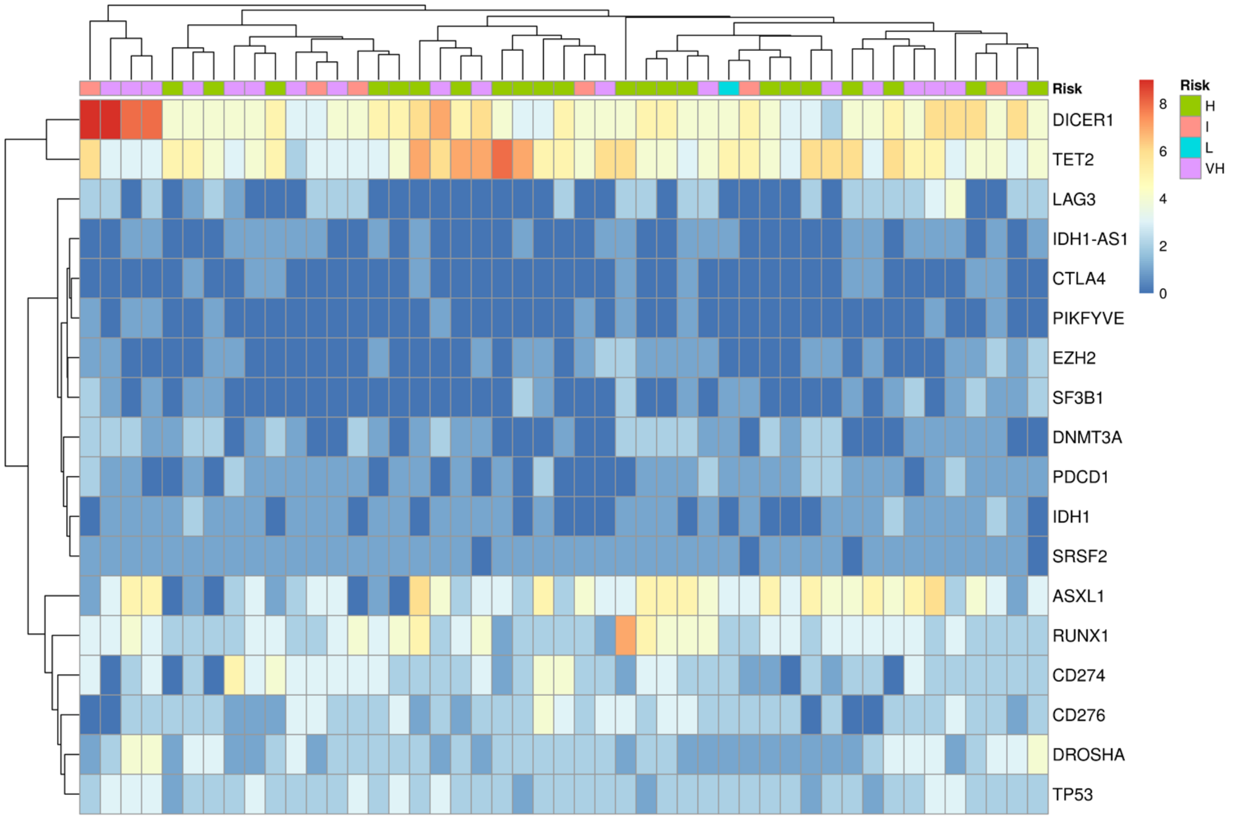

Supplement: S1 Fig — The brighter colors indicate higher number of mutations in the patients’ genes. The risk line is the IPSS-R score presented by groups: low (L), intermediate (I), high (H), very high (VH). (PDF) [file pone.0248430.s001.pdf]
