# Supplementary material for: High mutation burden in the checkpoint and micro-RNA processing genes in myelodysplastic syndrome
Source: PLoS One. 2021 Mar 17;16(3):e0248430. doi: 10.1371/journal.pone.0248430 (PMC7968630; doi:10.1371/journal.pone.0248430)

S2 Fig. Prevalence of common exonic pathogenic mutations leading to transcription failure. The left vertical bar indicate the percentage of patients with abnormalities in the gene. Upper horizontal bar characterize mutation burden in the individual patients per megabase pair. Middle boxplot indicates that type of mutations in the genes in individual patients. Mutations are ordered and presented based on their deleterious effect in the order as shown in the legend. Additional less deleterious mutations in the gene are not shown. Horizontal lower bar plot indicate the type of genetic alterations present in individual patient across all genes tested. The risk line is the IPSS-R score presented by groups: low (L), intermediate (I), high (H), very high (VH). Transplantation line indicates whether the patient was allografted.

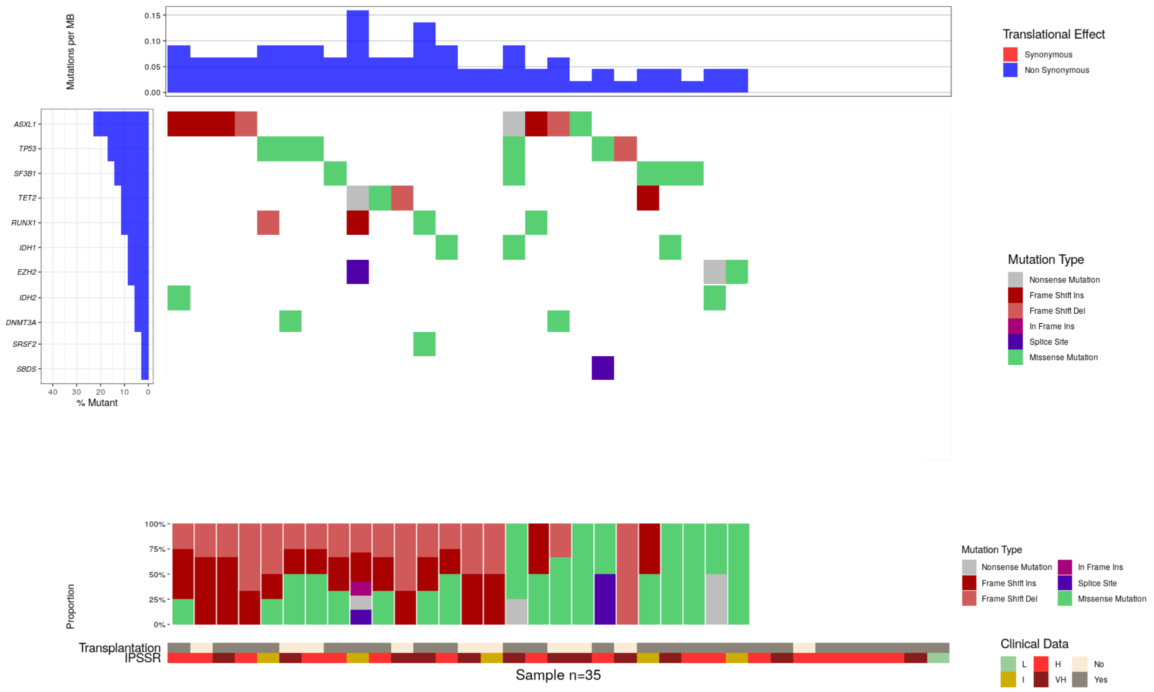

Supplement: S2 Fig — The left vertical bar indicate the percentage of patients with abnormalities in the gene. Upper horizontal bar characterize mutation burden in the individual patients per megabase pair. Middle boxplot indicates that type of mutations in the genes in individual patients. Mutations are ordered and presented based on their deleterious effect in the order as shown in the legend. Additional less deleterious mutations in the gene are not shown. Horizontal lower bar plot indicate the type of genetic alterations present in individual patient across all genes tested. The risk line is the IPSS-R score presented by groups: low (L), intermediate (I), high (H), very high (VH). Transplantation line indicates whether the patient was allografted. (PDF) [file pone.0248430.s002.pdf]
