# Supplementary material for: High mutation burden in the checkpoint and micro-RNA processing genes in myelodysplastic syndrome
Source: PLoS One. 2021 Mar 17;16(3):e0248430. doi: 10.1371/journal.pone.0248430 (PMC7968630; doi:10.1371/journal.pone.0248430)

S3 Fig. Cluster analyses and heatmap of mutations in the study group. The heat map includes both pathogeneic mutations and SNPs with undetermined significance. The risk line is the IPSS-R score presented by groups: low (L), intermediate (I), high (H), very high (VH). Dark blue color represents high allele frequency shift downwards (up to -100%) from common allele frequency in the sample (the most part of the sample is homozygous with presence of this SNP, while the particular patient is homozygous with absence of this SNP). Similarly, red colors represent high allele frequency allele frequency shift upwards (the most part of the sample is homozygous with absence of this SNP, while the particular patient is homozygous with presence of this SNP). Yellow colors represent allele frequencies close to the median allele frequency for this gene in a patient. The patients are clustered according to their mutation patterns.

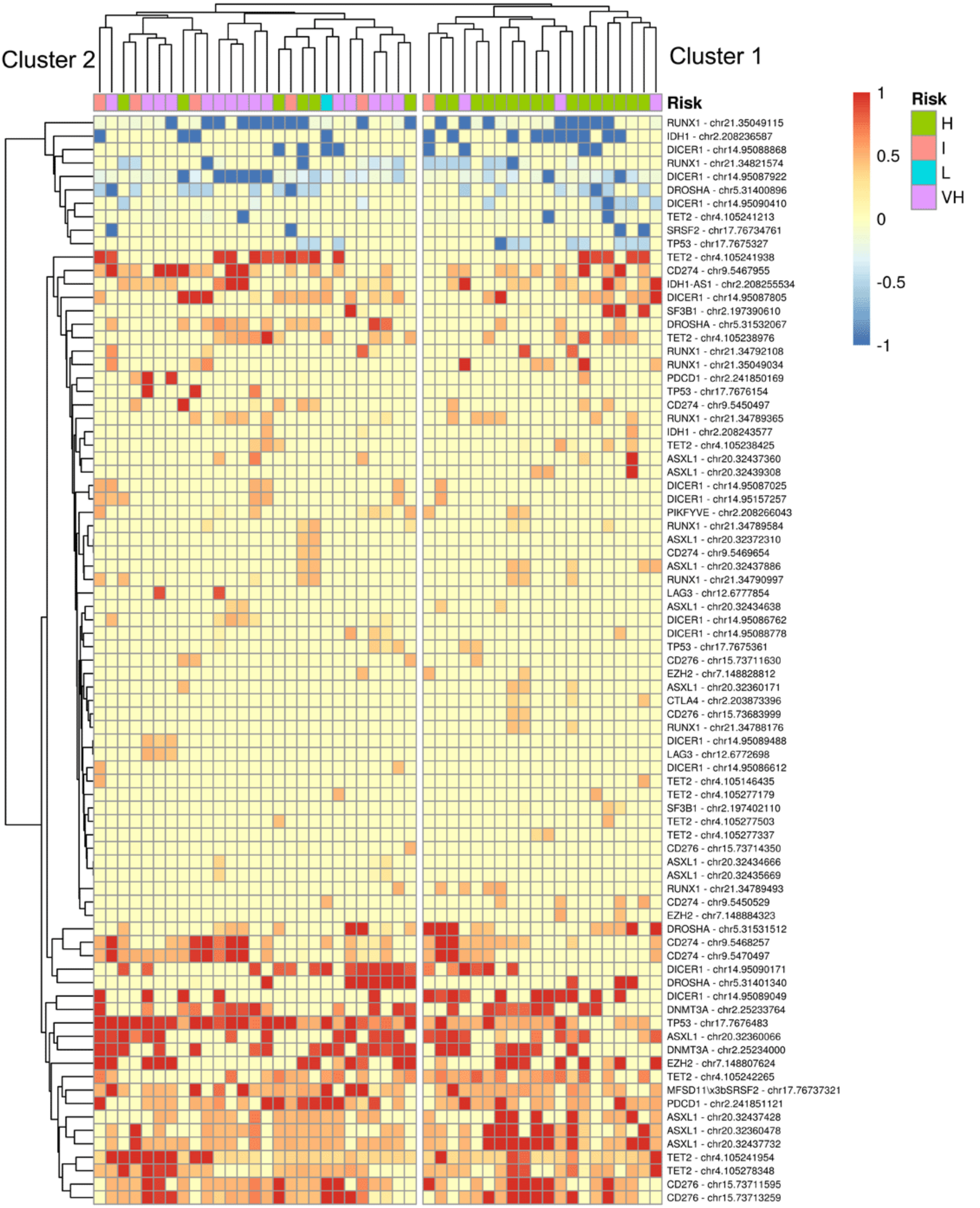

Supplement: S3 Fig — The heat map includes both pathogeneic mutations and SNPs with undetermined significance. The risk line is the IPSS-R score presented by groups: low (L), intermediate (I), high (H), very high (VH). Dark blue color represents high allele frequency shift downwards (up to -100%) from common allele frequency in the sample (the most part of the sample is homozygous with presence of this SNP, while the particular patient is homozygous with absence of this SNP). Similarly, red colors represent high allele frequency allele frequency shift upwards (the most part of the sample is homozygous with absence of this SNP, while the particular patient is homozygous with presence of this SNP). Yellow colors represent allele frequencies close to the median allele frequency for this gene in a patient. The patients are clustered according to their mutation patterns. (PDF) [file pone.0248430.s003.pdf]
