# Supplementary material for: High mutation burden in the checkpoint and micro-RNA processing genes in myelodysplastic syndrome
Source: PLoS One. 2021 Mar 17;16(3):e0248430. doi: 10.1371/journal.pone.0248430 (PMC7968630; doi:10.1371/journal.pone.0248430)

S4 Fig. Prevalence of exonic and UTR-3 SNPs in the studied gene panel. White color in the heatmap represents absence of mutations. Blue colors represent mutations with VAF<50%, orange and red with VAF>50%. The risk line is the IPSS-R score presented by groups: low (L), intermediate (I), high (H), very high (VH). Transplantation line indicates whether the patient was allografted. (*) indicates mutations which are present in the COSMIC database. (**) indicates mutations associated with oncohematological diseases in the COSMIC database.

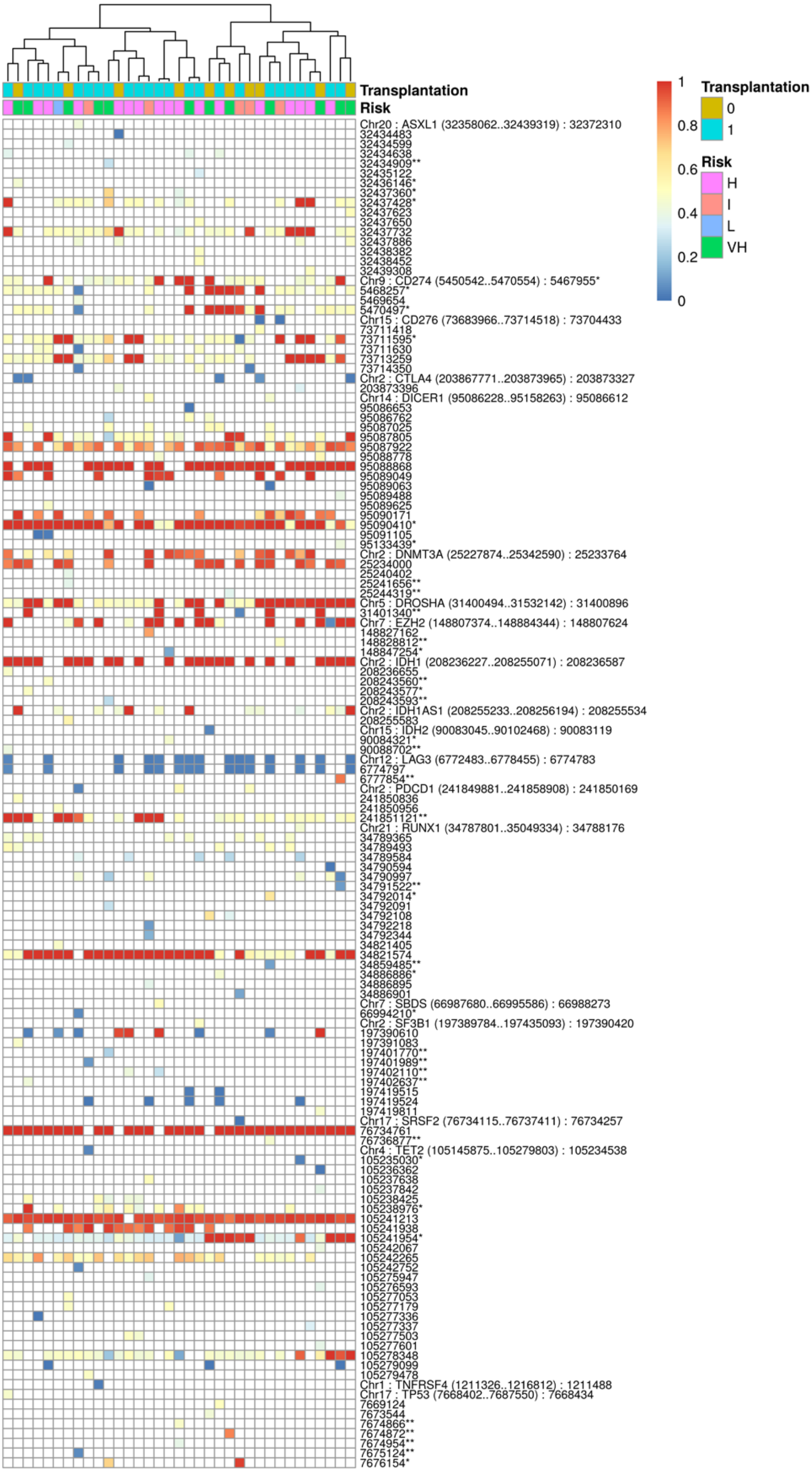

Supplement: S4 Fig — White color in the heatmap represents absence of mutations. Blue colors represent mutations with VAF<50%, orange and red with VAF>50%. The risk line is the IPSS-R score presented by groups: low (L), intermediate (I), high (H), very high (VH). Transplantation line indicates whether the patient was allografted. (*) indicates mutations which are present in the COSMIC database. (**) indicates mutations associated with oncohematological diseases in the COSMIC database. (PDF) [file pone.0248430.s004.pdf]
