# Supplementary figures and images for: High mutation burden in the checkpoint and micro-RNA processing genes in myelodysplastic syndrome
Source: PLoS One. 2021 Mar 17;16(3):e0248430. doi: 10.1371/journal.pone.0248430 (PMC7968630; doi:10.1371/journal.pone.0248430)

S5 Fig. Non-parametric analysis of clinical characteristics at diagnosis in clusters 1 and 2.

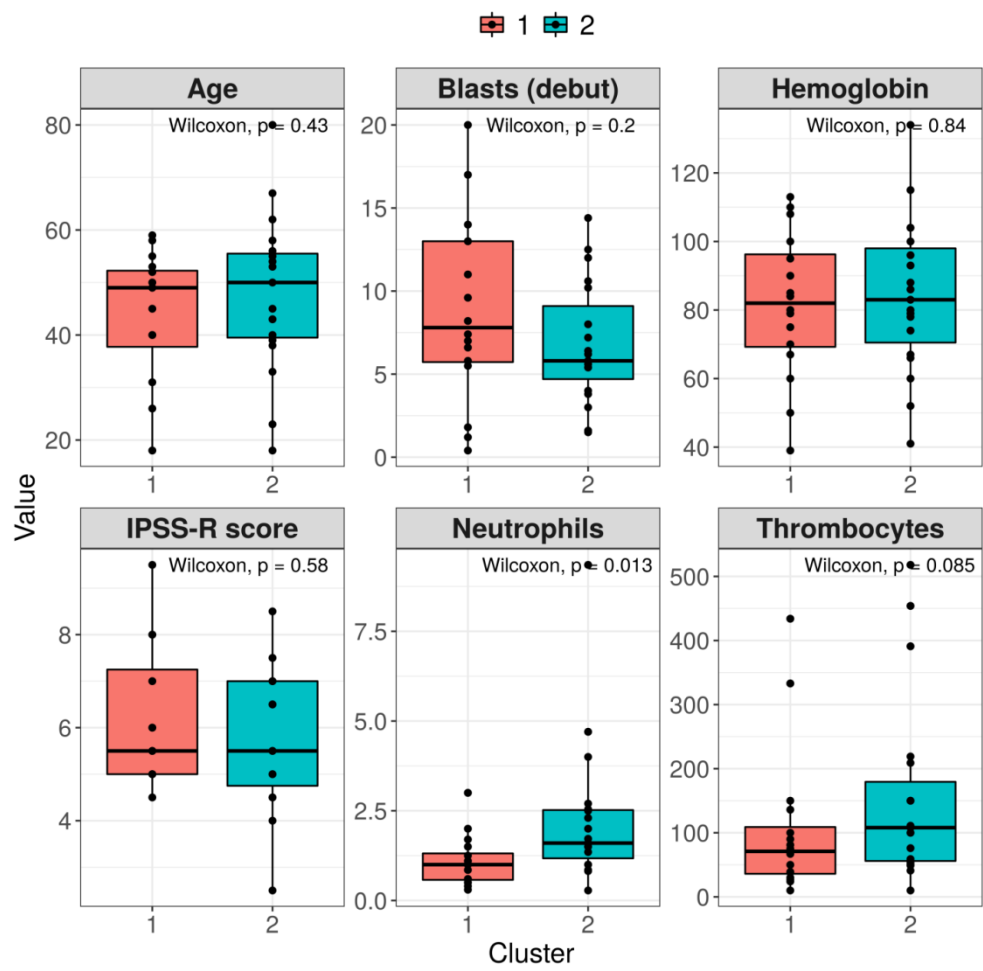

Supplement: S5 Fig — (PDF) [file pone.0248430.s005.pdf]

S7 Fig. Clonal evolution in the patients with longitudinal samples of bone marrow.

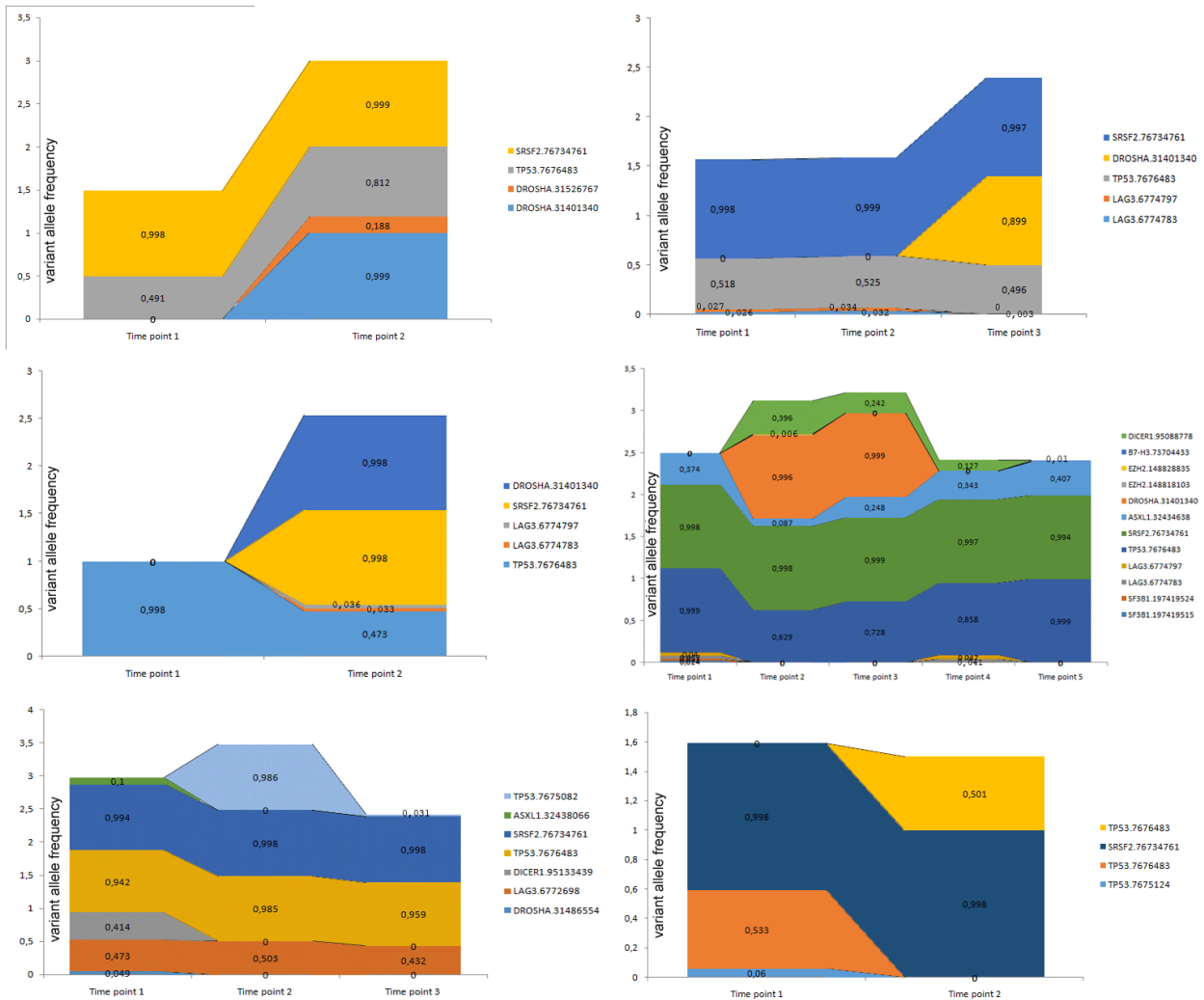

Supplement: S7 Fig — (PDF) [file pone.0248430.s007.pdf]
