# Supplementary material for: High mutation burden in the checkpoint and micro-RNA processing genes in myelodysplastic syndrome
Source: PLoS One. 2021 Mar 17;16(3):e0248430. doi: 10.1371/journal.pone.0248430 (PMC7968630; doi:10.1371/journal.pone.0248430)

S6 Fig. Associations between genetic abnormalities in the proportion of the study cohort with several mutated genes. Patients with only one mutated gene as well as genes with only one patient with mutation were excluded from analysis. The width of circus fragment represents the incidence of mutations in the specific genes. The width of the ribbon between the genes represents the rate of association.

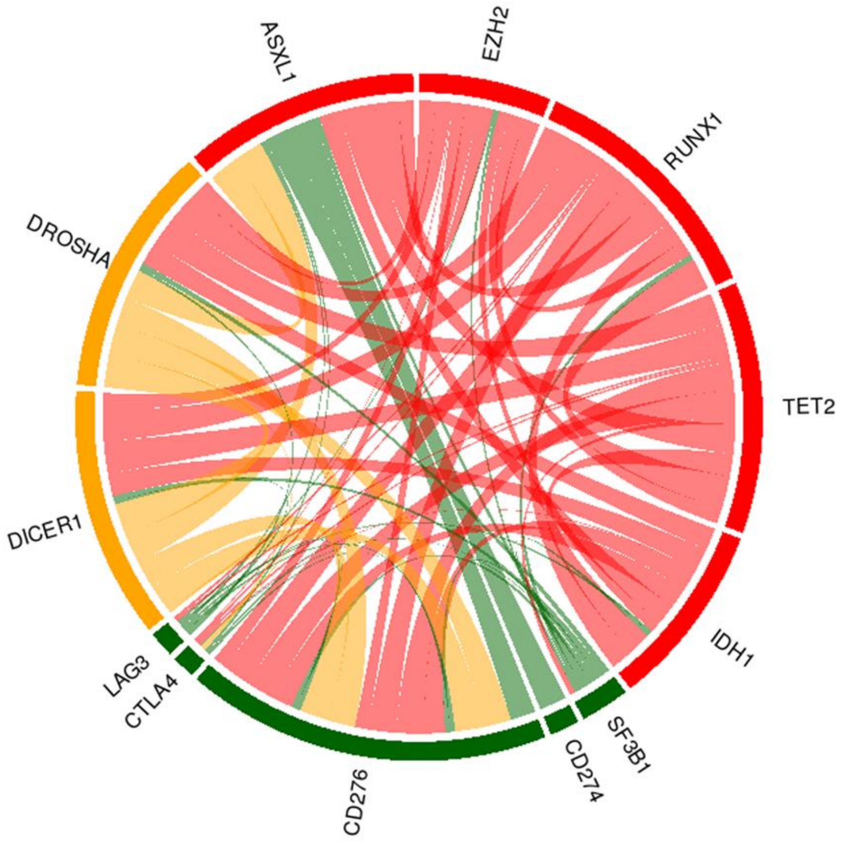

Supplement: S6 Fig — Patients with only one mutated gene as well as genes with only one patient with mutation were excluded from analysis. The width of circus fragment represents the incidence of mutations in the specific genes. The width of the ribbon between the genes represents the rate of association. (PDF) [file pone.0248430.s006.pdf]
